# Directed *In Vitro* Myogenesis of Human Embryonic Stem Cells and Their *In Vivo* Engraftment

**DOI:** 10.1371/journal.pone.0072023

**Published:** 2013-08-19

**Authors:** Yongsung Hwang, Samuel Suk, Susan Lin, Matthew Tierney, Bin Du, Timothy Seo, Aaron Mitchell, Alessandra Sacco, Shyni Varghese

**Affiliations:** 1 Department of Bioengineering, University of California San Diego, San Diego, California, United States of America; 2 Sanford Children’s Health Research Center, Sanford-Burnham Medical Research Institute, La Jolla, California, United States of America; 3 Department of Nanoengineering, University of California San Diego, San Diego, California, United States of America; University of Bristol, United Kingdom

## Abstract

Development of human embryonic stem cell (hESC)-based therapy requires derivation of *in vitro* expandable cell populations that can readily differentiate to specified cell types and engraft upon transplantation. Here, we report that hESCs can differentiate into skeletal muscle cells without genetic manipulation. This is achieved through the isolation of cells expressing a mesodermal marker, platelet-derived growth factor receptor-α (PDGFRA), following embryoid body (EB) formation. The ESC-derived cells differentiated into myoblasts *in vitro* as evident by upregulation of various myogenic genes, irrespective of the presence of serum in the medium. This result is further corroborated by the presence of sarcomeric myosin and desmin, markers for terminally differentiated cells. When transplanted *in vivo*, these pre-myogenically committed cells were viable in tibialis anterior muscles 14 days post-implantation. These hESC-derived cells, which readily undergo myogenic differentiation in culture medium containing serum, could be a viable cell source for skeletal muscle repair and tissue engineering to ameliorate various muscle wasting diseases.

## Introduction

Human embryonic stem cells (hESCs) hold great promise for treating various debilitating diseases due to their ability to proliferate indefinitely without phenotypic alterations and their capacity to differentiate into all cell types in the human body [1], [2], [3]. These qualities suggest widespread utility of hESCs in applications ranging from cell-based regenerative therapy to drug screening platforms [4], [5], [6]. Thus hESCs would be an ideal cell source for treating compromised muscle tissues.

Since the effective use of hESCs for regenerating injured or diseased skeletal muscle requires generation of a high-yield population of myogenic progenitors with proliferative and regenerative potential, considerable effort in derivation of such progenitors has been under way. Over the past decade, substantial strides have been made in generating muscle progenitor cells or terminally differentiated cells from ESCs and induced pluripotent stem cells (iPSCs) [7], [8], [9], [10]. This involves a variety of approaches ranging from mRNAs to proteins to small molecule treatment, although the most prominent and efficient methodology has thus far been genetic manipulation [11], [12], [13].

Darabi *et al.* have demonstrated that both ESCs and iPSCs can be successfully differentiated to myoblasts by forced expression of PAX3 or PAX7 transcription factors [14], [15]. Transplantation of pluripotent stem cell (PSC)-derived myogenic cells into damaged or degenerated muscles of *mdx* mice, a muscular dystrophy model, has been shown to contribute to tissue regeneration, albeit resulting in low engraftment efficiency [16], [17], [18]. Although genetic manipulation is an efficient strategy to direct differentiation of ESCs to targeted cellular phenotypes, from a therapeutic standpoint, directing differentiation without the need for introduction of transgenes is highly sought. Barberi *et al.* has demonstrated that myogenic precursors reside in CD73^+^/NCAM^+^ populations derived from hESCs and that these cells can engraft into muscles of SCID/Beige mice, suggesting the existence of myogenic progenitor cells within the hESC-derived mesoderm progenitor cells [19]. There also exist a number of other studies implying the ability of mesoderm progenitor cells derived from hESCs to undergo myogenic differentiation [15], [20], [21]. These findings indicate that hESC-derived myogenic cells could be an ideal cell source to treat compromised skeletal muscle tissues.

In this study, we examine the derivation of progenitor cells that exhibit the ability to differentiate into myoblasts from hESCs without genetic manipulation. We also investigate the *in vivo* engraftment of these ESC-derived cells into skeletal muscle of NOD/SCID mice.

## Materials and Methods

### Maintenance of Human Embryonic Stem Cells

The OCT4-GFP reporter line was generated as described earlier [22]. The HUES9-OCT4-GFP cells were expanded on MEFs (mouse embryonic fibroblasts) using Knockout DMEM supplemented with 10% KSR (knockout serum replacement), 10% human plasmanate (Talecris Biotherapeutics), 1% NEAA (non-essential amino acids), 1% penicillin/streptomycin, 1% Gluta-MAX, and 55 µM 2-mercaptoethanol as described elsewhere [22]. The cells were enzymatically (Accutase; Millipore) passaged when they reached ∼80% confluency and were supplemented with fresh medium containing 30 ng/ml of bFGF (Life Technologies) daily.

### Derivation of PDGFRA^+^ Cells

The undifferentiated HUES9-OCT4-GFP cell colonies were enzymatically detached from MEFs and dissociated into single cells by incubating with Accutase for 5 mins. Roughly 1.0×10^6^ cells were suspended in high glucose DMEM containing 5% FBS, 2 mM L-glutamine, 100 nM dexamethasone, 100 µM hydrocortisone, 1% penicillin/streptomycin, 1 mM transferrin, 86.1 µM recombinant insulin, 2 µM progesterone, 10.01 mM putrescine, and 3.01 µM selenite (Life Technologies). The cells were cultured in suspension by using ultra low attachment plates in a 37°C/5% CO_2_ incubator to form embryoid bodies (EBs) for 9 days. The medium was changed every other day. The EBs were split at a ratio of 1∶6, transferred to a 10 cm dish coated with growth factor-reduced Matrigel (1∶25 in KnockOut DMEM; BD Biosciences), and cultured using the aforementioned medium. The cells were adhered onto the Matrigel-coated dishes 24 hrs after plating and cultured for an additional 7 days until a significant number of migrating cells from EBs was observed. The cells growing out of the EBs were dissociated by trypsin and filtered using a cell strainer with a pore size of 40 µm. The isolated cells were then concentrated for PDGFRA^+^/OCT4-GFP^−^ and PDGFRA^−/^OCT4-GFP^−^ cell populations by fluorescence-activated cell sorting (FACS). The PDGFRA^+^ and PDGFRA^−^ cells were then expanded in growth medium (high glucose DMEM containing 10% FBS, 2 mM L-glutamine, and 1% penicillin/streptomycin) before characterizing them for their differentiation potential.

### FACS Analysis

The cells migrating out of the EBs on Matrigel-coated dishes were dissociated with Accutase and resuspended in a buffer solution (2% FBS/0.09% sodium azide/DPBS; BD Biosciences) and stained directly with Alexa-647-conjugated PDGFRA or Alexa Fluor 647 mouse IgM,κ isotype control antibodies (Biolegend). Cells were stained for 30 mins on ice, washed, and resuspended in a buffer solution. Samples were analyzed by using BD Biosystems FACSCanto. Data were analyzed with the CellQuest Pro software.

### Population Doubling Time

Population doubling time (PDT) of PDGFRA^+^ cell populations while undergoing myogenic differentiation either in serum-containing medium or serum-free medium was calculated using the equation below [23]:
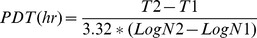
where T1 and T2 represent days 3 and 5, respectively; N1 and N2 are the number of cells at T1 and T2, respectively. For PDT measurement and proliferation profile, PDGFRA^+^ cells were detached and dissociated into single cells by using Accutase, and the number of cells was counted using a hemocytometer.

### Myogenic Differentiation

To achieve myogenic differentiation, PDGFRA^+^ and PDGFRA^−^ cells (after 4 passages) were plated onto tissue culture plates at an initial cell density of 1×10^4^ cells/cm^2^ and cultured in high glucose DMEM containing 2 mM L-glutamine, 100 nM dexamethasone, 100 µM hydrocortisone, 1% penicillin/streptomycin, 1 mM transferrin, 86.1 µM recombinant insulin, 2 µM progesterone, 10.01 mM putrescine, and 3.01 µM selenite with 10% FBS or without FBS. The myogenic differentiation potential of PDGFRA^+^ cells was further confirmed with cells after passage 8.

### Osteogenic Differentiation

To induce osteogenic differentiation in monolayer culture, hESC-derived PDGFRA^+^ cells were plated at an initial cell density of 1×10^4^ cells/cm^2^ in osteogenic medium containing high glucose DMEM, 10% FBS, 50 µg/ml ascorbic acid, 10 mM β-glycerophosphate, 100 nM dexamethasone, and 1% penicillin/streptomycin for up to 14 days.

### Adipogenic Differentiation

Adipogenic differentiation was achieved by culturing hESC-derived PDGFRA^+^ cells plated at an initial cell density of 1×10^4^ cells/cm^2^ in adipogenic medium containing high glucose DMEM, 10% FBS, 1 µg/ml human recombinant insulin, 0.5 mM 3-isobutyl-1-methylxanthine, 10 µM dexamethasone, and 1% penicillin/streptomycin for up to 14 days.

### Chondrogenic Differentiation

For chondrogenic differentiation, 3×10^5^ hESC-derived PDGFRA^+^ cells were collected in 15 mL conical tubes and centrifuged at 1000 rpm for 5 mins to form pellets. The pellets were cultured in chondrogenic medium containing high glucose DMEM, 2 mM L-glutamine, 100 nM dexamethasone, 50 mg/mL ascorbic acid phosphate, 1 mM sodium pyruvate, 40 mg/mL proline, 1% ITS (insulin, transferrin, and selenous acid), 1% penicillin/streptomycin, and 10 ng/mL transforming growth factor (TGF-β1) for up to 21 days.

### Immunofluorescent Staining

Immunofluorescent staining was performed using the following primary antibodies: PAX3, PAX7, MF20 (1∶200; Developmental Studies Hybridoma Bank), MYOD, MYF5 (1∶200; Santa Cruz Biotechnology), desmin (1∶200; Abcam), human lamin A/C (1∶50; Vector Laboratories), and mouse laminin (1∶200; Millipore). The following secondary antibodies were used: goat anti-rat Alexa 546 (1∶200; Life Technologies), goat anti-mouse Alexa488 (1∶250; Life Technologies), and goat anti-rabbit Alexa 546 (1∶200; Life Technologies). For immunofluorescent staining of cells grown on tissue culture plates, cells were fixed in 4% PFA for 10 min at room temperature. Immediately before staining, the cells were permeabilized with 0.1% (v/v) Triton X-100 and blocked with 3% (w/v) BSA for 30 mins. Cells were stained with primary antibodies diluted in 1% BSA overnight at 4°C, washed 3 times with PBS, and incubated with secondary antibodies for 1 hr at room temperature. The nuclei were stained with Hoechst 33342 (2 µg/ml; Life Technologies) for 5 mins at room temperature. For immunofluorescent staining of tibialis anterior (TA) muscles, samples were first embedded in optimal temperature cutting compound (OCT) for cryosectioning and sections (having around 10 µm thickness) were fixed with 4% PFA for 10 mins at room temperature. Next, sections were permeabilized with 0.3% Triton X-100, blocked with 20% goat serum for 1 hr at room temperature, and stained with human lamin A/C. For antigen retrieval, the sections stained with human lamin A/C were immersed in preheated (90°C) 10 mM citric acid (pH 6) for 15 mins and rinsed with PBS three times prior to incubating with PAX7 antibody followed by incubating with secondary antibody for 1 hr at room temperature. Imaging was performed using a fluorescence microscope (Carl Zeiss; Axio Observer A1).

### Cell Shape Analyses

Cell alignment and orientation for PDGFRA^+^ cells grown in serum-containing and serum-free media were calculated using NIH ImageJ software as previously reported. Figure S1 provides a detailed schematic 24,25,26. Briefly, alignment angles of the nuclei were calculated based on the orientation of the major elliptic axis of each nucleus with respect to the horizontal axis, followed by renormalizing these angles to the mean orientation angle of all nuclei. The frequency of alignment angles was plotted in 10 degree increments to their mean orientation. To investigate the elongation of nuclei, the nuclear shape index was calculated as circularity (4π × Area/perimeter^2^) and aspect ratio (ratio of major axis to minor axis of each nucleus). The differentiation index was estimated as the ratio of MF20-positive nuclei to the total number of nuclei and the fusion index was calculated by dividing the total number of nuclei in multinucleated myotubes (≥2 nuclei) with the total number of MF20-positive nuclei [27]. The calculations were made by analyzing images of cells stained for MF20 using Macbiophotonics ImageJ software (McMaster University, Ontario). The number of MF20-positive cells was counted manually. For counting the nuclei, the images were filtered and adjusted for threshold followed by calculating the total number of nuclei (i.e., DAPI-positive cells). Four random fields of view were analyzed per sample for each of the three independent experiments.

### Alkaline Phosphatase (ALP) Staining

ALP staining was performed using Sigma Kit #85L-2 (Sigma-Aldrich) by following the manufacturer’s instructions [28]. In brief, cells were fixed in acetone/citrate solution, rinsed with water, and stained with Fast Blue RR/naphthol for 30 mins at room temperature.

### Alizarin Red S Staining

Cells were fixed with 4% paraformaldehyde prior to staining, and fixed cells were incubated for 30 mins at room temperature in a 40 mM Alizarin Red S solution (pH 4.1) followed by washing with PBS to remove unincorporated dye.

### RNA Isolation and Quantitative PCR

Total RNA was extracted using TRIzol (Invitrogen), and reverse transcription was performed using iScript cDNA synthesis kit (BioRad) by following manufacturer’s protocols. Quantitative PCR was carried out by using SYBR Select Master Mix (Life Technologies) and the ABI Prism 7300 Sequence Detection System (Applied Biosystems). Gene expression was normalized to 18S rRNA levels as reference and delta Ct values were calculated as C_t_^target^ – C_t_^18s^. All experiments were performed with three biological replicates and the relative fold changes were calculated as 2^−ΔΔCt^ [29]. The PCR primers used in this study are listed in Table S1.

### Cell Transplantation

24 hours prior to cell transplantation, the TA muscles of 2-month-old immune-deficient NOD.CB17-Prkdc^scid^/J mice (NOD/SCID mice) were injured using cardiotoxin. The mice were first anesthetized by injecting ketamine (100 mg/kg) and xylazine (10 mg/kg) intraperitoneally and then injected with 25 µL (0.5 mg/mL) of cardiotoxin (Sigma, Cat# c9759) into the TA muscle. Using a 28 Gauge syringe, two hESC-derived PDGFRA^+^ (5.0×10^5^ cells) cell populations of varying myogenic commitment were suspended in 10 µL of physiological saline solution and injected into the TA muscles with and without cardiotoxin injury. All animal studies were carried out with the approval of Institutional Animal Care and Use Committee (IACUC) of the University of California, San Diego. Two weeks (14 days) following transplantation, muscles were harvested and the *in vivo* viability of the donor cells was assessed histologically.

### Statistical Analysis

All values were presented as mean ± standard deviation and statistical significance was determined by two-tailed unpaired Student’s t-test or single-factor analysis of variance (ANOVA) with Tukey’s Multiple Comparison Test (*p<0.05 and **p<0.01).

## Results

### Derivation of PDGFRA^+^ Myogenic Progenitors from hESCs

**Figure 1:**
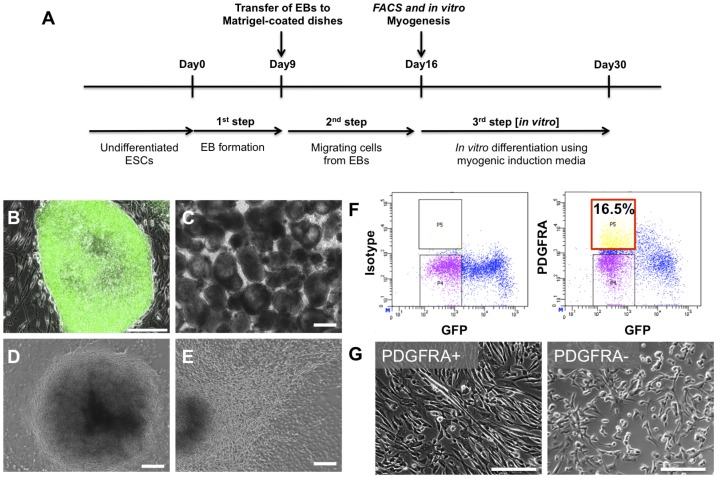
Derivation of PDGFRA^+^ myogenic progenitors from hESCs. (**A**) Schematic depicting the isolation protocol for PDGFRA^+^ cells. (**B**) Undifferentiated hESC colony showing OCT4-GFP expression. (**C**) EB formation. (**D**) EB attached to the Matrigel-coated tissue culture plates. (**E**) Migration of cells from EBs. (**F**) FACS demonstrating isolation of PDGFRA^+^/OCT4-GFP^−^ population. (**G**) Cells attached to 0.1% gelatin-coated tissue culture plates sorted against PDGFRA^+^ (left) and PDGFRA^−^ (right). Scale bar = 200 µm.

Figure 1 summarizes the derivation of PDGFRA^+^ cell population from HUES9 cells. The EBs of HUES9-OCT4-GFP cells were formed via suspension culture in medium containing 2 mM L-glutamine, 100 nM dexamethasone, 100 µM hydrocortisone, 1% penicillin/streptomycin, 1 mM transferrin, 86.1 µM recombinant insulin, 2 µM progesterone, 10.01 mM putrescine, and 3.01 µM selenite with 5% FBS (Figs. 1B,C). The EBs were then plated onto Matrigel-coated tissue culture plates to facilitate adhesion. The migration of cells from EBs was evident 2–3 days post-plating (Figs. 1D,E). The spindle-shaped cells migrating out of the EBs were mechanically isolated and purified for PDGFRA^+^ cells. Since we have used an OCT4-GFP reporter cell line, the isolated cells were purified for a PDGFRA^+^/OCT4-GFP^−^ cell population, where OCT4 is a marker for undifferentiated ESCs (Fig. 1F). Figure 1G shows the gross morphology of PDGFRA^+^/OCT4-GFP^−^ (PDGFRA^+^) and PDGFRA^−/^OCT4-GFP^−^ (PDGFRA^−^) cell population in monolayer culture. Furthermore, the PDGFRA^+^ cells expressed undetectable levels of NANOG by qPCR (Fig. S2), indicating absence of pluripotent ESCs within the isolated cells.

### Myogenic Differentiation of PDGFRA^+^ Cells

**Figure 2:**
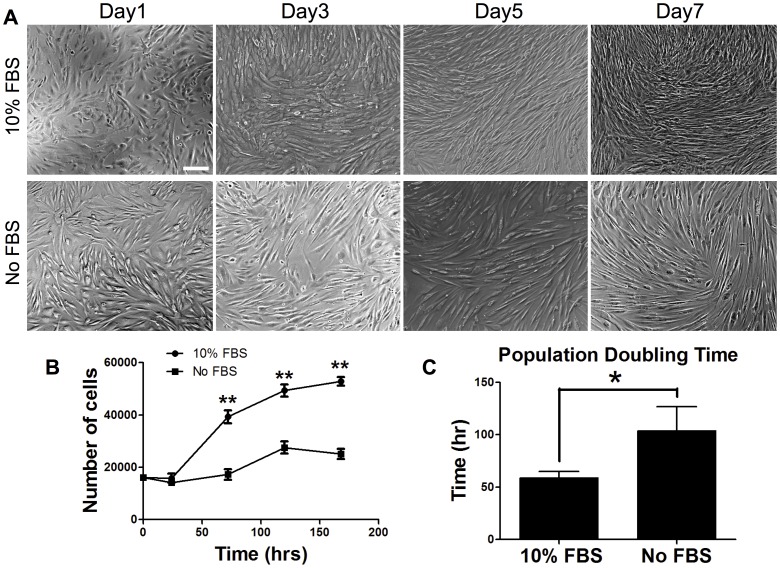
Characterization of the proliferative potential of PDGFRA^+^ cells. (**A**) Phase contrast images of PDGFRA^+^ cells undergoing myogenic differentiation at different time points (top: 10% FBS, bottom: No FBS). (**B**) Proliferation profiles of cells grown in serum-containing and serum-free media. (**C**) Population doubling time. Scale bar = 200 µm. *p<0.05 and **p<0.01.

**Figure 3:**
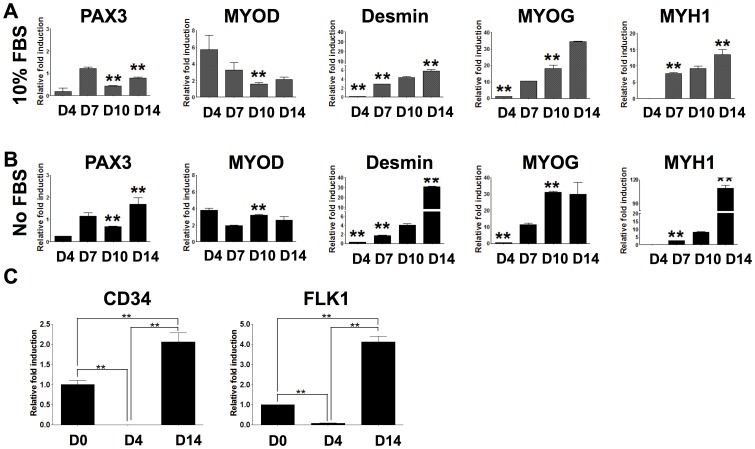
*In vitro* myogenic differentiation potential demonstrated by quantitative PCR. Gene expression profiles of PDGFRA^+^ cells as a function of culture in serum-containing (**A**) and serum-free (**B**) media. Statistical analysis was performed between serum-containing (A) and serum-free (B) myogenic media for each corresponding time point. **p<0.01. (**C**) PDGFRA^+^ cells undergoing myogenic differentiation show upregulation of endothelial markers. **p<0.01.

**Figure 4:**
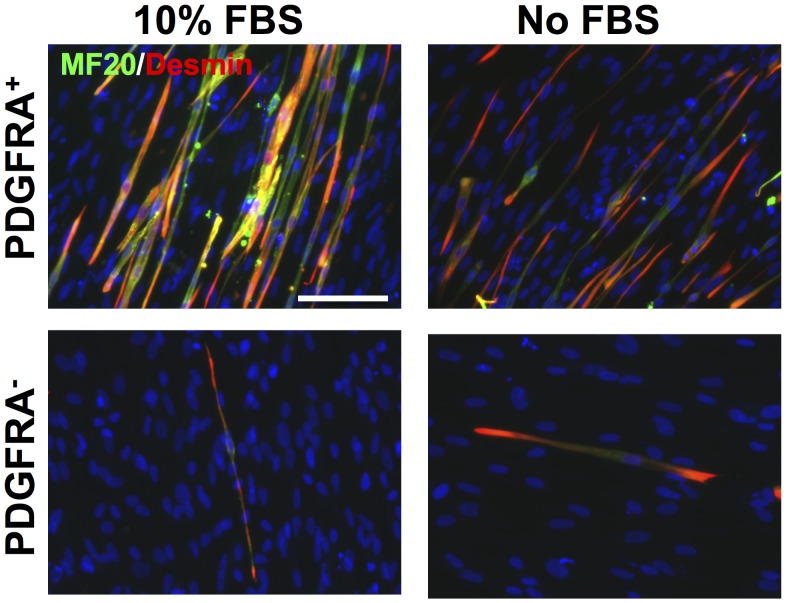
Terminal myogenic differentiation characterized by immunofluorescent staining. Immunofluorescent staining for MF20 (green) and desmin (red) of PDGFRA^+^ (top) PDGFRA^−^ (bottom) cells grown in serum-containing (left) and serum-free (right) media. Scale bar = 100 µm.

Defining and establishing optimal culture conditions for acquiring a large number of myogenic progenitors with high proliferative and differentiation capacity is an essential step toward clinical use of hESCs for muscle tissue repair and regeneration. Therefore, the myogenic differentiation potential of hESC-derived PDGFRA^+^ cells was evaluated *in vitro* using either serum-containing or serum-free media. In a time course experiment, we have observed that the morphology of PDGFRA^+^ cells progressively became more spindle-like in both media types (Fig. 2A). As anticipated, the PDGFRA^+^ cells cultured in serum-free medium showed cell death and reduced proliferation compared to serum-containing media (Figs. 2B,C). Interestingly, as evident from the gene expression profile, the PDGFRA^+^ cells underwent myogenic differentiation irrespective of the presence of serum in the medium (Figs. 3A,B). In addition to the myogenic markers, the cells also exhibited an upregulation of other markers such as CD34 and FLK1 (Fig. 3C). The cells stained positive for MF20 (sarcomeric myosin) and desmin (muscle-specific intermediate filament), further corroborating the gene expression profile (Fig. 4). The differentiating cells were able to fuse and form multinucleated myotubes. In contrast, little to no myogenic differentiation was observed in PDGFRA^−^ cell population under identical culture conditions (Figs. 4, S3).

### Cell Shape Analyses of PDGFRA^+^ Cells Undergoing Myogenic Differentiation

**Figure 5:**
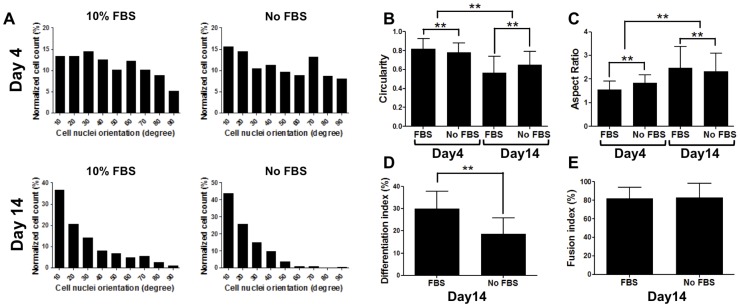
Cell shape analyses of PDGFRA^+^ cells undergoing myogenic differentiation. (**A**) Alignment and orientation of PDGFRA^+^ cells grown in serum-containing (left) and serum-free (right) media. (**B, C**) Shape indices for PDGFRA^+^ cells while undergoing terminal myogenic differentiation in serum-containing (left bar) and serum-free (right bar) media. n = 794, 385, 425, and 210, respectively. (D) Estimated differentiation indices of PDGFRA^+^ in serum and serum-free media. (E) Estimated fusion indices of differentiated PDGFRA^+^ (MF20 positive cells) in serum and serum -free media. n = 722 and 245, respectively. **p<0.01.

We performed morphological analysis to determine the changes in cellular shape as the PDGFRA^+^ cells differentiated into myoblasts. As shown in Fig. 5A, myogenic progenitor cells in the presence or absence of serum were randomly oriented at day 4. However, when they underwent terminal myogenic differentiation (at day 14), cells were highly oriented and aligned. Similarly, we have calculated various shape indexes including circularity, aspect ratio, and fusion indices. Figure S1 shows morphological changes of cell nuclei with culture time. At early stages of myogenic differentiation, cell nuclei were found to be more spherical, which is represented as circularity, and had lower aspect ratio (Fig. S1). However, upon terminal myogenic differentiation, the cell nuclei became more elongated and had higher aspect ratios for both serum-containing and serum-free media (Figs. 5B,C). In addition to the nuclei shape, we also calculated the differentiation indices to assess differentiation efficiency and the fusion indices for fully differentiated multinucleated myotubes, demonstrating that there were more differentiated cells in cultures containing serum compared to serum-free medium (Fig. 5D, E).

### *In vitro* Mesodermal Differentiation Potential of hESC-derived PDGFRA^+^ Cell Population

**Figure 6:**
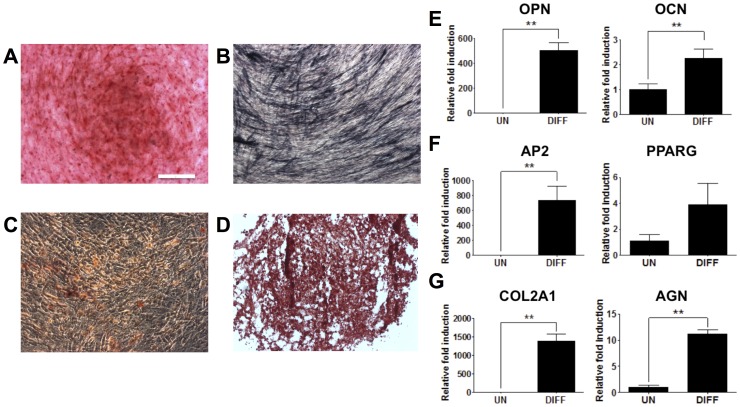
*In vitro* mesodermal differentiation of PDGFRA^+^ cells. Osteogenic differentiation as characterized by staining for Alizarin Red S (**A**), alkaline phosphatase (**B**), and gene expression (**E**). Adipogenic differentiation as characterized by Oil Red O staining (**C**) and gene expression (**F**). Chondrogenic differentiation as characterized by Safranin O staining (**D**) and gene expression (**G**). UN, undifferentiated PDGFRA^+^ cells cultured in growth medium; DIFF, PDGFRA^+^ cells cultured in differentiation inducing medium. Scale bar = 200 µm. **p<0.01.

Next, we characterized the mesodermal differentiation potential of hESC-derived PDGFRA^+^ cells into osteogenic, adipogenic, and chondrogenic lineages. As shown in Fig. 6, the PDGFRA^+^ cells underwent osteogenic, chondrogenic, and adipogenic differentiation. Osteogenic differentiation was confirmed by positive staining for Alizarin Red S (Fig. 6A) and alkaline phosphatase (Fig. 6B), and upregulation of osteogenic markers such as osteopontin (OPN) and osteocalcin (OCN) (Fig. 6E). Adipogenic differentiation was evaluated by the presence of lipid production shown by Oil Red O staining (Fig. 6C) and by upregulated gene expressions for, adipocyte fatty acid binding protein 2 (AP2) and peroxisome proliferator-activated receptor-γ (PPARG) (Fig. 6F). The cells also underwent chondrogenic differentiation in pellet culture. The pellets stained positive for Safranin O (Fig. 6D) and exhibited upregulation of chondrogenic markers, such as collagen type II and aggrecan (Fig. 6G).

### *In vivo* Engraftment of hESC-derived Myogenic Progenitors

**Figure 7:**
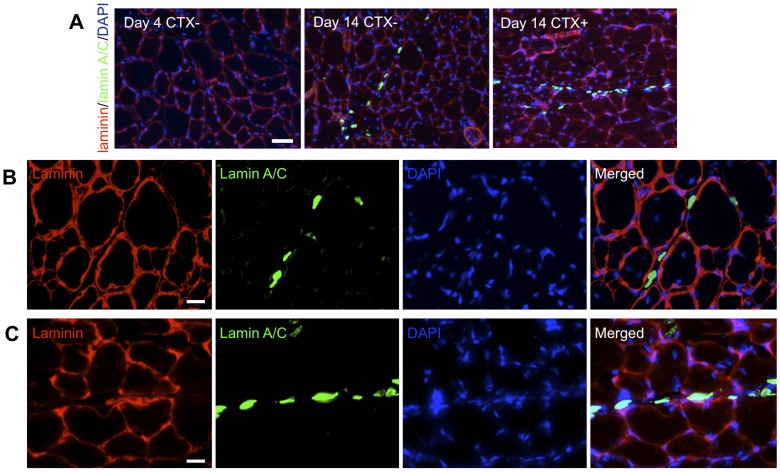
Engraftment of myogenic progenitors in NOD/SCID mice. (**A**) Immunofluorescent staining of TA muscle sections from NOD/SCID mice injected with cells grown for 4 days (left) and 14 days with (CTX+) and without cardiotoxin injury (CTX-) for mouse laminin (red), human lamin A/C (green), and nuclei (blue). Corresponding high magnification images for muscles treated with cells cultured for 14 day without (**B**) and with (**C**) cardiotoxin injury. Scale bar = 50 µm, 20 µm, and 20 µm, repectively.

Having established their myogenic differentiation potential, we next determined the *in vivo* engraftment potential of the hESC-derived myogenic progenitor cells. Since the culture medium containing serum did not have any adverse effect on myogenic differentiation of PDGFRA^+^ cells, we used cells cultured in serum media for the *in vivo* studies as serum-containing medium yields higher cell number. For *in vivo* cell transplantation, we have used two different cell populations with varying degrees of myogenic commitment – cells cultured for 4 days and 14 days. Unlike the cells cultured for 14 days, the PDGFRA^+^ cells cultured for 4 days did not stain positive for MF20 or desmin (data not shown). The cells were transplanted into TA muscles of 2-month-old immune-deficient NOD/SCID mice with or without cardiotoxin injury. Two weeks after transplantation, TA muscles were collected and assessed for donor cell engraftment by immunostaining with human lamin A/C, and mouse laminin antibodies (Figure 7). As seen in figure 7A, we did not observe any viable donor cells in mice injected with cells cultured for 4 days prior to *in vivo* transplantation. On the other hand, we have detected positive staining of human-specific nuclei in mice injected with pre-myogenically committed cells (14 days cultured cells) (Figs. 7A–C).

## Discussion

There has been remarkable progress in the field of hESC differentiation research in the last decade [30], [31], [32]. Since its isolation [1], its seemingly unlimited self-renewal and differentiation capacity has garnered considerable attention; such characteristics present opportunities to utilize hESCs as a cell source for regenerative medicine and drug screening platforms. Beyond the potential for hESCs to be used in cell therapy, recent work in hESC research has provided promising evidence for its utility in developmental biology. Some laboratories have already used hESCs as a platform for studying signaling cues important in proliferation and differentiation in the early embryo [33].

Muscle impairment from trauma, tumor recession, or degenerative muscle diseases such as muscular dystrophies represents a significant clinical problem. Cell transplantation has been considered to be one of the most promising therapies for compromised muscle tissues [34], [35], [36], [37]. HESCs could be an ideal cell source for treating muscle disease as they could offer a large number of progenitor cells for replenishing lost tissues and compromised cells. Despite the explosion in hESC differentiation research, differentiation of hESCs into skeletal muscle cells is sparse and often requires genetic manipulation [11], [12], [14], [15]. One of the obvious strategies is to derive progenitor cells with the ability to undergo myogenic differentiation. Previously, we and others have shown that mesoderm progenitor cells with multi-potentiality and *in vivo* tissue-forming abilities can be derived from hESCs *via* EB formation [38], [39], [40]. A number of studies have shown that the hESC-derived mesoderm progenitor cells contain a small population of cells with the ability to undergo myogenic differentiation potential [19], [20]. In the present work we determined the myogenic differentiation potential of PDGFRA^+^ cells derived from hESCs.

We show that the PDGFRA^+^ cells derived from hESCs through EB formation and cultured in induction medium can undergo terminal differentiation into myocytes and fuse to form myotubes *in vitro*. The progenitors were able to retain differentiation capacity despite numerous passaging (∼8 passages). The hESC-derived PDGFRA^+^ cells underwent myogenic differentiation irrespective of the presence of serum in the culture medium. The fact that the progenitor cells exhibit myogenic differentiation in serum-containing medium offers a benefit in that our derivation can produce high cell numbers without compromising yield of the targeted cell type, which is a key necessity for realizing cell-based therapies. The PDGFRA^+^ cells differentiated into the myogenic lineage *in vitro* exhibiting upregulation of FLK1 could play a significant role in promoting angiogenesis by recruiting endothelial cells and thereby enhancing function and viability of the transplanted cells [41]. Previous studies have shown the importance of vascularization for the viability and function of the transplanted cells and cell-laden scaffolds and how co-expression of endothelial markers such as FLK1 could significantly improve the *in vivo* viability of donor cells [38], [42], [43].

In addition to myogenic differentiation, hESC-derived PDGFRA^+^ cells were also able to undergo osteogenic, chondrogenic, and adipogenic differentiation *in vitro* when cultured in medium containing corresponding differentiation-inducing soluble factors. This is not surprising as most progenitor cells, including satellite cells, have been shown to undergo differentiation into cell types of other mesodermal lineages *in vitro* responding to soluble cues [38], [44], [45]. In a recent study, Awaya *et al*., have shown that hPSC-derived progenitor cells can successfully differentiate into myoblasts *in vitro* [40]. These *in vitro*-engineered myoblasts contributed to tissue repair when implanted into cardiotoxin-injured muscles.

Our *in vivo* data suggests that the amount of culture time and the differentiation state of the cells before transplantation is of utmost importance in ensuring a viable and engraftable population. The cells cultured *in vitro* for 4 days prior to transplantation were unable to engraft into muscles of NOD/SCID recipient mice, whereas cells cultured for 14 days *in vitro* to pre-commit to the myogenic lineage engrafted into the host tissue, suggesting that degree of commitment may be a key consideration for implantation. In addition to differences in myogenic expression levels, day 14 cells exhibited higher levels of CD34 and FLK1 expression. A number of studies have shown the importance of these markers on *in vivo* myogenic differentiation of transplanted cells [46], [47]. CD34 expression has also been shown to play a key role in satellite cell function. For instance, studies by Alfaro *et al*. have shown that CD34 expression promotes satellite cell proliferation and their ability to contribute to skeletal muscle regeneration [48]. It remains unknown whether the stage dependency is the main factor, or if it depends on environmental influences prior to implantation. Correlating the stage specificity of differentiated cells to their engraftment efficiency is beyond the scope of this study.

In conclusion, this study provides a proof-of-concept that engraftable myogenic progenitor cells can be generated from hESCs without the need for genetic manipulation. The engrafted cells did not contribute to teratoma formation, consistent with previous studies that showed the potential of *in vivo* transplanted cells to contribute to tissue repair without teratoma formation [49]. Such ESC-derived cells would have significant impact in treating various muscle injuries and degenerative diseases such as muscular dystrophies.

## Supporting Information

Figure S1 **Schematics of cell shape analyses.** Schematic depicting the cell shape analyses with representative images for cell nuclei at days 4 and 14 in culture.
(TIFF)

Figure S2 **Analysis of pluripotency of PDGFRA^+^ cells.** Gene expression level of NANOG, a pluripotent marker, for undifferentiated HUES9, PDGFRA^+^ cells (day 0), and PDGFRA^+^ cells grown in serum-containing medium for 14 days. **p<0.01.
(TIFF)

Figure S3 **Comparisons of** ***in vitro*** **myogenic differentiation potential between PDGFRA^+^ and PDGFRA^−^** **cells.** Quantitative PCR analysis showing cell-specific differences in gene expression levels of PDGFRA^+^ cells (A^+^) and PDGFRA^−^ cells (A^−^) grown in serum-containing media for 14 days. *p<0.05 and **p<0.01.
(TIFF)

Table S1 **List of primers used for quantitative PCR.**
(TIFF)
