# Correction: Directed *In Vitro* Myogenesis of Human Embryonic Stem Cells and Their *In Vivo* Engraftment

**DOI:** 10.1371/annotation/b02313dc-840f-4f03-91a2-77cb55a3a4c9

**Published:** 2013-09-18

**Authors:** Yongsung Hwang, Samuel Suk, Susan Lin, Matthew Tierney, Bin Du, Timothy Seo, Aaron Mitchell, Alessandra Sacco, Shyni Varghese

1. On Page 2, Derivation of PDGFRA+ Cells, it should be 10 µM transferrin, 860.9 nM recombinant insulin, 20 nM 

 progesterone, 100.1 µM putrescine, and 30.1 nM selenite. 

2. Affiliation of Sacco A should be Sanford Children’s Health Research Center, Sanford- Burnham Medical Research 

 Institute, La Jolla, California, United States of America

3. Additional Acknowledgments: We would like to acknowledge the Developmental Studies Hybridoma Bank for providing the PAX3 antibody generated by Ordahl Lab, the PAX7 antibody generated by Kawakami Lab, and the MF20 antibody generated by Fischman Lab. These antibodies are developed under the auspices of the NICHD and maintained by The University of Iowa, Department of Biology, Iowa City, IA 52242.

