# Supplementary figures and images for: Directed In Vitro Myogenesis of Human Embryonic Stem Cells and Their In Vivo Engraftment
Source: PLoS One. 2013 Aug 19;8(8):e72023. doi: 10.1371/journal.pone.0072023 (PMC3747108; doi:10.1371/journal.pone.0072023)

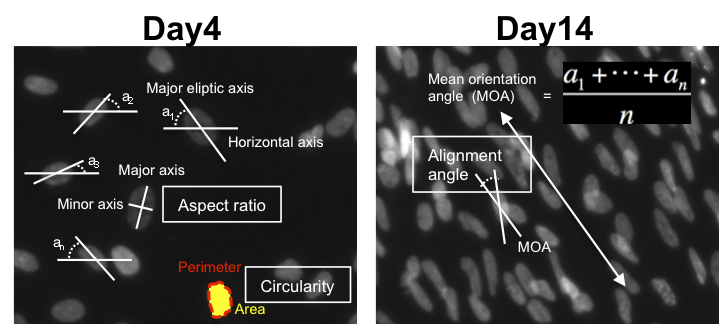

Supplement: Figure S1 — Schematics of cell shape analyses. Schematic depicting the cell shape analyses with representative images for cell nuclei at days 4 and 14 in culture. (TIFF) [file pone.0072023.s001.tiff]

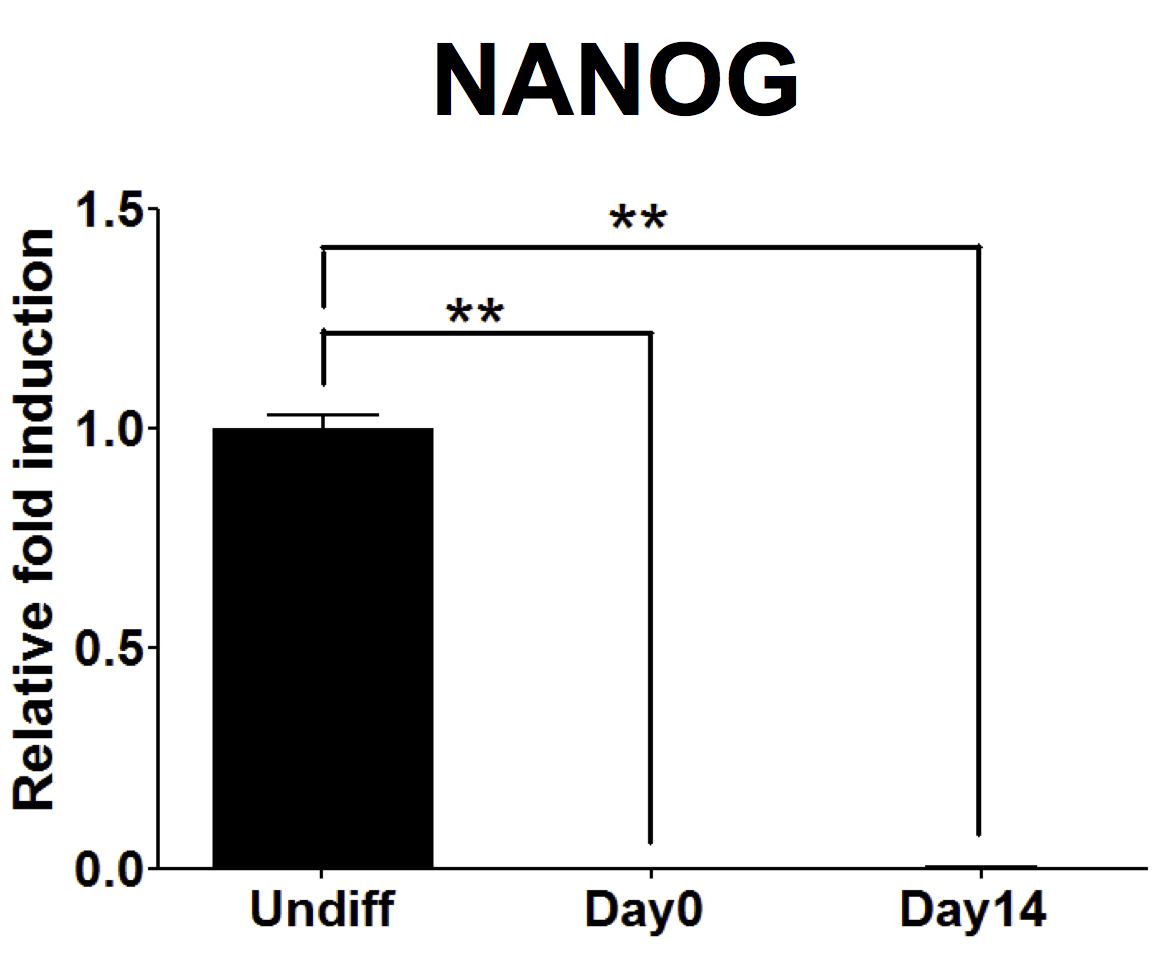

Supplement: Figure S2 — Analysis of pluripotency of PDGFRA+ cells. Gene expression level of NANOG, a pluripotent marker, for undifferentiated HUES9, PDGFRA+ cells (day 0), and PDGFRA+ cells grown in serum-containing medium for 14 days. **p<0.01. (TIFF) [file pone.0072023.s002.tiff]

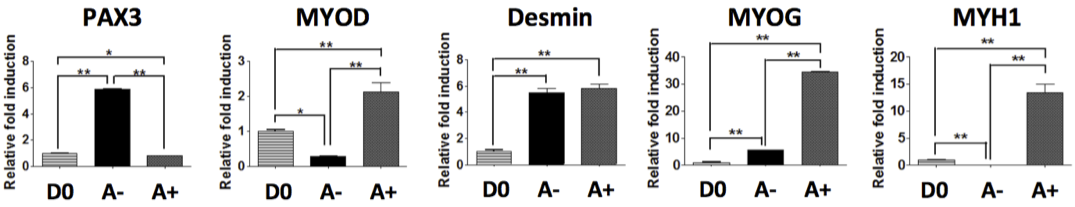

Supplement: Figure S3 — Comparisons of in vitro myogenic differentiation potential between PDGFRA+ and PDGFRA− cells. Quantitative PCR analysis showing cell-specific differences in gene expression levels of PDGFRA+ cells (A+) and PDGFRA− cells (A−) grown in serum-containing media for 14 days. *p<0.05 and **p<0.01. (TIFF) [file pone.0072023.s003.tiff]
